# Apoptotic and Anti-Inflammatory Effects of* Eupatorium japonicum* Thunb. in Rheumatoid Arthritis Fibroblast-Like Synoviocytes

**DOI:** 10.1155/2018/1383697

**Published:** 2018-07-09

**Authors:** Jong-Il Shin, Yong-Joon Jeon, Sol Lee, Yoon Gyeong Lee, Ji Beom Kim, Hak Cheol Kwon, Sung Hun Kim, Inki Kim, Kyungho Lee, Ye Sun Han

**Affiliations:** ^1^Department of Biological Sciences, Konkuk University, 120 Neungdong-ro, Gwangjin-gu, Seoul 05029, Republic of Korea; ^2^Korea Institute of Science and Technology (KIST), Gangneung, Gangwondo 25451, Republic of Korea; ^3^Department of Convergence Medicine, Asan Institute for Life Sciences, Asan Medical Center, Seoul 05505, Republic of Korea; ^4^Hemp Institute, Konkuk University, 120 Neungdong-ro, Gwangjin-gu, Seoul 05029, Republic of Korea; ^5^Department of Advanced Technology Fusion, Konkuk University, 120 Neungdong-ro, Gwangjin-gu, Seoul 05029, Republic of Korea

## Abstract

Rheumatoid arthritis (RA) is a chronic autoimmune inflammatory disease characterized by synovitis, hyperplasia, and the destruction of bone and cartilage. A variety of immunosuppressive biological agents have been developed because the pathogenesis of RA is related predominantly to the inflammatory response. However, rheumatoid arthritis fibroblast-like synovial cells (RAFLS), which are known to play an important role in RA progression, exhibit resistance to immunosuppressants through cancer-like properties. In this study, we identified a novel therapeutic compound for RA, which reduced inflammation and the abnormal proliferation of RAFLS in natural product library made from Korean native plants.* Eupatorium japonicum* Thunb. (EJT) extract, a component of the natural product library, most effectively reduced viability through the induction of ROS-mediated apoptosis in a dose-dependent manner. In addition, the increased ROS induced the expression of ATF4 and CHOP, key players in ER stress-mediated apoptosis. Interestingly, EJT extract treatment dose-dependently reduced the expression of IL-1*β* and the transcription of MMP-9, which were induced by TNF-*α* treatment, through the inhibition of NF-*κ*B and p38 activation. Collectively, we found that EJT extract exerted apoptotic effects through increases in ROS production and CHOP expression and exerted anti-inflammatory effects through the suppression of NF-*κ*B activation, IL-1*β* expression, and MMP-9 transcription.

## 1. Introduction

Rheumatoid arthritis (RA) is a chronic systemic inflammatory disease of unknown etiology [1]. Recent studies have suggested that genetic and environmental factors influence the development and progression of RA [2, 3]. RA is characterized by synovitis, hyperplasia, and the destruction of bone and cartilage [1]. The increased proliferation and insufficient apoptosis of macrophage-like synovial cells (MLS) and fibroblast-like synoviocytes (FLS), which constitute synovium, contribute to synovial membrane hyperplasia [4, 5]. It was reported that rheumatoid arthritis fibroblast-like synoviocytes (RAFLS) were key players in the joint destruction process and progression of RA [6]. RAFLS are activated by inflammatory cytokines, particularly by TNF-*α* and IL-1*β*, and produce matrix-degrading proteases such as matrix metalloproteinases (MMPs). In addition, RAFLS form a paracrine and autocrine network with MLS and this interaction contributes to chronic synovitis [7]. As the pathogenesis of RA is primarily associated with the inflammatory response, several immunosuppressive biologic agents have been developed. However, the success of these biological agents is limited by uncertain pathophysiological responses and other side effects. In addition, the cancer-like characteristics of RAFLS show resistance to immunosuppressive agents [5]. Therefore, it is very important to find a new therapeutic agent that targets the heterogeneous features of RAFLS.

In the synovium and synoviocytes of patients with RA, endoplasmic reticulum (ER) stress-related gene expression is strongly increased. In addition, GRP78, induced by proinflammatory cytokines, enhances the survival and proliferation of synoviocytes [8]. ER stress is induced by the accumulation of misfolded or unfolded proteins in the ER lumen under insufficient protein folding conditions. The mechanism to resolve the accumulation of unfolded proteins in the ER lumen is called the unfolded protein response (UPR). When ER stress is not rectified through the UPR, apoptosis is initiated to remove unhealthy cells [9, 10]. C/EBP homologous protein (CHOP), a proapoptotic transcription factor induced by ATF4 and ATF6, is key player in ER stress-mediated apoptosis [10]. CHOP contributes to apoptosis through the downregulation of the expression of Bcl-2 and the upregulation of DR5 expression [11, 12]. In addition, ATF4 and CHOP lead to oxidative stress and apoptosis through an increase in gene transcription associated with protein synthesis [13].

Natural products have emerged as a major source of drug development. Natural product-based drugs, including natural products or synthetic compounds based on natural product, have been approved for the treatment of a variety of therapeutic indications, such as anticancer, anti-infective, and antidiabetic effects [14, 15]. In this study, the developed methodology of high-throughput screening (HTS) enables screening for substances that show an apoptotic effect of RAFLS from established natural products.

*Eupatorium japonicum* is a Chinese medicinal herb. The leaves and stems are effective painkillers, antivirals, diuretics, carminatives, antibacterials, and vermifuges. The leaves and stems are used for the treatment of nausea, vomiting, indigestion, and diarrhea [16]. The extract of* E. japonicum* contains the essential oil thymol and several pyrrolizidine alkaloids (PAs); the PAs of the* E. japonicum* extract are known to include indicine, amabiline, viridiflorine, echinatine, and rinderine [17]. The WHO report and other studies have demonstrated the hepatotoxic and carcinogenic effects of PAs [18–20]. However, it has also been reported that indicine-N-oxide, derived from indicine, exerts anticancer effects through the DNA damage and microtubules depolarization [21]. However, the effects of* E. japonicum* extract on the inflammatory response and RA have not been reported.

In this study, we investigated the antirheumatoid effects of EJT extract and the underlying molecular mechanism in MH7A cells (RAFLS). We showed that EJT extract induced apoptosis and ER stress through ROS-dependent manner. In addition, CHOP, a key player in ER stress-mediated apoptosis, was involved in EJT extract-mediated apoptosis. EJT extract also decreased the expression of MMP-9 and IL-1*β* through the inhibition of NF-*κ*B activation.

## 2. Materials and Methods

The natural product library was provided by Korea Institute of Science and Technology (KIST; Gangneung, Korea). The extract library was composed of ethanolic extracts of Korean native plants. MTT (tetrazolium bromide), dimethyl sulfoxide (DMSO), and N-Acetyl-L-Cysteine (NAC) were purchased from Sigma (St. Louis, MO). 2′,7′-Dichlorofluorescein diacetate (DCF-DA) was purchased from Molecular Probes (Eugene, OR). The TNF-*α* was purchased from Peprotech (Rocky Hill, NJ). The anti-PARP-1, anti-ATF4, anti-caspase-7, anti-actin, and anti-GAPDH antibodies were purchased from Santa Cruz Biotechnology (Santa Cruz, CA). The anti-CHOP, anti-phospho-NF-*κ*B, anti-NF-*κ*B, anti-IL-1*β*, anti-phospho-p38, anti-p38, anti-phospho-JNK, anti-JNK, anti-phospho-eIF2*α*, anti-eIF2*α*, and horseradish peroxidase-conjugated secondary antibodies were purchased from Cell Signaling Technology (Beverly, MA). Annexin V-flamma 488 was purchased from Bioacts (Korea). Hoechst 33342 was purchased from Invitrogen (CA).

### 2.1. Cell Lines and Cell Culture

The MH7A cells, immortalized human rheumatoid arthritis fibroblast-like synoviocytes (hRAFLS) with SV40 T antigen [22], were grown in RPMI-1640 medium (WelGENE, Daegu, Korea) supplemented with 10% heat-inactivated fetal bovine serum (Biowest, Nuaille, France) and 1% penicillin/streptomycin mixture (Gibco) under a humidified atmosphere in 5% CO_2_ at 37°C. HEK293 cells were grown in DMEM (WelGENE) supplemented with 10% heat-inactivated fetal bovine serum (Biowest) and 1% penicillin/streptomycin mixture (Gibco).

### 2.2. Hairpin Design and shRNA Preparation

One shRNA pair was chosen according to CHOP (NM_001109986.1) sequences following the Addgene (Cambridge, MA) guidelines. Each sequence contained a specific 21 nucleotide rat CHOP sense sequence followed by a short space (CTCGAG), an antisense 21 nucleotides sequence, and a transcription termination signal. Target sequences for CHOP shRNA are 5′-CCGGGAGGAAGAAGAGGAAGATCAA*CTCGAG*TTGATCTTCCTCTTCTTCCTCTTTTTG-3′. The shRNA pair, designed to contain terminal* Eco*RI and* Age*I restriction sites, was subcloned into a vector after annealing to generate the pLKO.1-CHOP shRNA vector.

### 2.3. Lentiviral Production and Lentiviral Infection

Lentiviruses were produced by transfection of HEK293 cells with a four-plasmid system containing pLKO.1-shRNA, pMDLg/pRRE, pMD2-VSVG, and pRSV-Rev. The transfection was performed with iN-fect (iNtron, Korea) in accordance with the manufacturer's instructions. Lentiviral supernatants were collected at 24 and 48 h posttransfection and filtered. The filtered lentivirus supernatants were stored in a deep freezer at -70°C. MH7A cells were plated in 100-mm cell culture dishes (0.8 × 10^6^ cells/dish). After 16 h, the cells were infected with 1 ml lentivirus supernatants with 8 *μ*g/ml polybrene. At approximately 24 h postinfection, the media was replaced with media supplemented with 0.8 *μ*g/ml puromycin. After 48 h, puromycin-containing media was replaced with normal growth media for 24 h; subsequently, the puromycin selection was repeated again. After selection, the content of CHOP protein was measured by immunoblot analysis of the soluble protein isolated from each cell lines.

### 2.4. Measurement of Cell Viability

MTT was used to determine the relative number of viable cells. MH7A cells were seeded into a 24-well cell culture plate and incubated for 16 h. The cells were treated with various concentrations of natural product extracts. If required, the cells were pretreated with ROS scavenger NAC for 2 h before EJT extract treatment. After the cells were washed twice with PBS, 300 *μ*l or 500 *μ*l of fresh medium containing 5 *μ*g/ml MTT was added to each well and incubated for 2 h at 37°C. The formazan precipitate was dissolved in 400 *μ*l DMSO and the absorbance of solution substrate was measured at 570 nm by using an ELISA Reader (UYM 340; ASYS Hitech, Salzburg, Austria).

### 2.5. Annexin V Staining and Analysis

MH7A cells (0.05 × 10^6^ cells or 0.01 × 10^6^ cells) were plated in a confocal dish or confocal 96-well cell culture plate, respectively. After 16 h, the cells were treated with various concentrations of natural product extracts. After treatment with the natural product extract for 16 h, MH7A cells were washed once with PBS and then washed once with 1× binding buffer [20 mM Hepes (pH 7.4), 150 mM NaCl, and 2.5 mM CaCl_2_]. MH7A cells were stained with 1× binding buffer containing Annexin V (1 : 200) and Hoechst (1 : 5000). After 15 min, MH7A cells were washed twice with PBS. The intensity of Annexin V and Hoechst staining was detected by a superresolution confocal laser scanning microscope (LSM 800, Carl Zeiss) using 488 nm or 350 nm for excitation (magnification 400×). Annexin V intensity in the cell membrane region was analyzed by using the Operetta high-content analysis system (Perkin Elmer, MA). The Operetta high-content analysis system provided confocal images in specified areas for each well. The acquired images were analyzed by the Harmony software and provided statistically significant data.

### 2.6. Immunoblot Analysis

The cells were harvested in NET lysis buffer [150 mM NaCl, 1% NP-40, 50 mM Tris-HCl (pH 7.4), 2.5 mM EDTA] supplemented with 1% phosphatase, protease, and proteasome inhibitors. The protein concentration was quantified by using the Bradford method (Bio-Rad, Hercules, CA). Proteins were boiled in 1× sample buffer [500 mM Tris-HCl (pH 6.8), 10% SDS, 20% glycerol, 0.05% bromophenol blue, and 1%  *β*-mercaptoethanol] for 10 min at 100°C and were separated on SDS-polyacrylamide gels. The separated proteins were electrotransferred to Immobilon-P membranes (Millipore Corp., Bedford, MA, USA) and blotted with the indicated antibodies at 4°C overnight in Tris-buffered saline containing 0.08% Tween 20 (TBST) and 1% nonfat milk. The membranes were then incubated with horseradish peroxidase-conjugated antibodies at 25°C (room temperature) for 2 h, and the band signal was detected by using an LAS-4000 Luminescent Image Analyzer (Fujifilm, Tokyo, Japan). To confirm the equal loading of samples, the blots were stripped in stripping buffer [100 mM *β*-mercaptoethanol, 2% SDS, and 62.5 mM Tris-HCl (pH 6.8)] at 60°C for 30 min, washed three times with TBST buffer for 10 min each, and reprobed with other specific antibodies.

### 2.7. Detection of Intracellular ROS Levels

Intracellular ROS production was detected by using DCF-DA as an intracellular fluorescence probe. Briefly, the cells were treated with 20 *μ*M DCF-DA for 2 h at 37°C. When required, the ROS scavenger NAC was cotreated with EJT extract. The extract was added in each well. The cells were incubated for 6 h and the fluorescence intensity of DCF was detected by a fluorescence microscope (ECLIPSE TS-200; Nikon) using an excitation of 488 nm (magnification 200×).

### 2.8. Flow Cytometry Analysis of Intracellular ROS Levels

Briefly, MH7A cells were treated with EJT extract for 6 h and then treated with 20 *μ*M DCF-DA for 30 min at 37°C. The fluorescence intensity of DCF was determined by using flow cytometry (CytoFLEX, Beckman Coulter).

### 2.9. Real-Time Quantitative PCR

Real-time quantitative PCR was preformed using HiPi Real-time PCR 2 × Master Mix (SYBR Green, ELPiS, Korea) with 40 cycles. The cycle threshold (CT) was observed to extension step. Analysis of melting curve was carried out to convict specific amplification.

### 2.10. Statistical Analysis

The figures in this study are representative of a minimum of three independent experiments. All results were shown as the mean ± SD The statistical significance of the data between the experimental groups was evaluated by Student's* t*-test. A* P* value of less than 0.05 was considered to be statistically significant.

## 3. Results

### 3.1. High-Throughput Screening Assays Showing Apoptotic Effect in Natural Product Library

**Figure 1:**
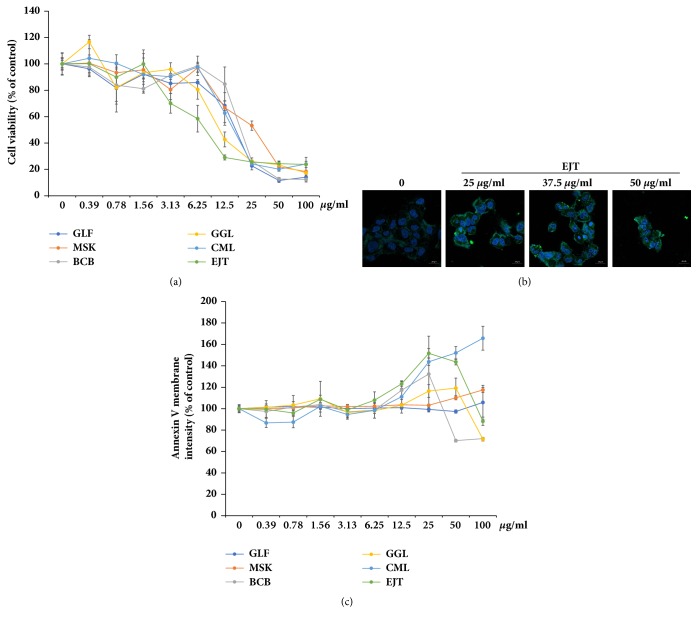
Screening of substances with apoptotic effects in the natural product library. (a) MH7A cells were treated with the indicated concentrations of natural product extracts for 24 h. The cell viability was determined by using MTT assay. Compounds GLF, MSK, BCB, GGL, CML, and EJT, corresponding to* Glehnia littoralis *Fr. Schm*., Magnolia sieboldii *K. Koch*., Boswellia carterii *Birdw*., Gomphrena globose *L*., Chelidonium majus *L., and* Eupatorium japonicum *Thunb. (b) MH7A cells were treated with the indicated concentration of EJT extract for 12 h and then stained with Annexin V 488 (green) and Hoechst 33342 (blue) at 25°C (room temperature) for 30 min. The staining intensity of Annexin V in the membrane was observed by confocal laser scanning microscope (magnification 400×). (c) Annexin V intensity in the cell membrane region was analyzed by using the Operetta high-content imaging system.

One characteristic of RA is synovial hyperplasia caused by inflammation and the uncontrolled proliferation of synovial fibroblasts [4]. Therefore, we tried to find new materials that reduced the abnormal proliferation of RA fibroblast-like synoviocytes (MH7A cells) from a natural product library. First, MH7A cells were treated with the indicated concentration (0.39–100 *μ*g/ml) of each natural product extract for 24 h and natural product extracts that significantly reduced the viability of MH7A cells to below 50% at a final concentration below 50 *μ*g/ml were identified. Six of the 430 screened natural product extracts reduced the viability of MH7A cells in a dose-dependent manner (Figure 1(a)).

We clarified whether the decrease in cell viability after treatment with the natural product extracts resulted from apoptotic effects through the use of Annexin V staining. When* E. japonicum* Thunb. (EJT) extract was treated at a concentration of 25–50 *μ*g/ml, Annexin V staining intensity was increased in the cell membrane of MH7A cells (Figure 1(b)). To compare the apoptotic effects of the natural product extracts and select the most effective compounds, we measured the Annexin V intensity in the cell membrane region by using the Operetta high-content analysis system. The Operetta high-content analysis system collects confocal images and analyzes the acquired images to provide statistically significant data. As shown in Figure 1(c),* Chelidonium majus L.* (CML) and EJT extract increased the intensity of Annexin V in a dose-dependent manner. As the EJT extract reduced viability to a greater extent than CML extract (Figure 1(a)) and induced apoptosis in MH7A cells, we conducted further tests on the EJT extract.

### 3.2. EJT Extract Induces ROS-Dependent Apoptosis in MH7A Cells

**Figure 2:**
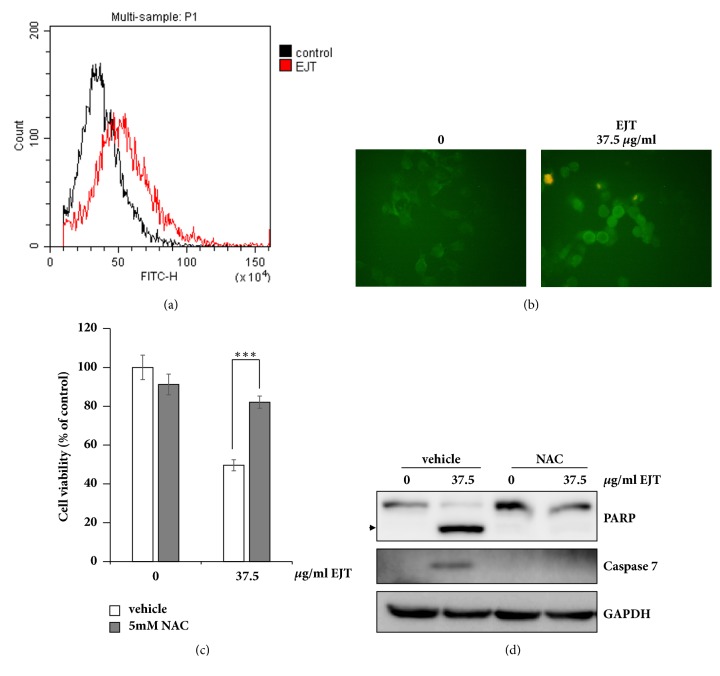
EJT extract induced ROS-dependent apoptosis in MH7A cells. (a) MH7A cells were treated with 37.5 *μ*g/ml EJT extract for 6 h and then treated with 20 *μ*M DCF-DA for 30 min. The intensity of intracellular ROS was detected by using flow cytometry. (b) MH7A cells were pretreated with 20 *μ*M DCF-DA for 2 h and then treated with 37.5 *μ*g/ml EJT extract for 6 h. The intensity of intracellular ROS was observed by using a fluorescence microscope (magnification 400×). (c) MH7A cells were pretreated with 5 mM NAC for 2 h and then treated with 37.5 *μ*g/ml EJT extract for 24 h. Cell viability was determined by using the MTT assay. Significant differences were indicated as ^*∗∗∗*^p < 0.001. (d) The cell lysates were subjected to SDS-PAGE and the separated samples were analyzed by immunoblotting for antibodies specific to PARP, caspase 7, and GAPDH.

To determine whether the apoptotic effects of EJT extract were associated with oxidative stress, we measured ROS generation by using the cell permeant oxidation-sensitive dye 2′,7′-dichlorofluorescein diacetate (DCF-DA). EJT extract treatment significantly increased ROS generation in MH7A cells (Figures 2(a) and 2(b)). Further, we investigated whether the increased generation of ROS after EJT extract treatment was related to apoptotic effects. It was observed that pretreatment of N-Acetyl-L-Cysteine (NAC), a ROS scavenger, significantly rescued viability reduced by EJT extract treatment (Figure 2(c)). In addition, the activation of caspase-7 and cleavage of PARP by EJT extract were significantly reduced by pretreatment of NAC (Figure 2(d)). These results indicated that pretreatment of NAC inhibited the apoptosis induced by EJT extract treatment and further suggested that apoptosis induced by EJT extract was dependent on ROS generation.

### 3.3. CHOP Is Involved in EJT Extract-Mediated Apoptosis in MH7A Cells

**Figure 3:**
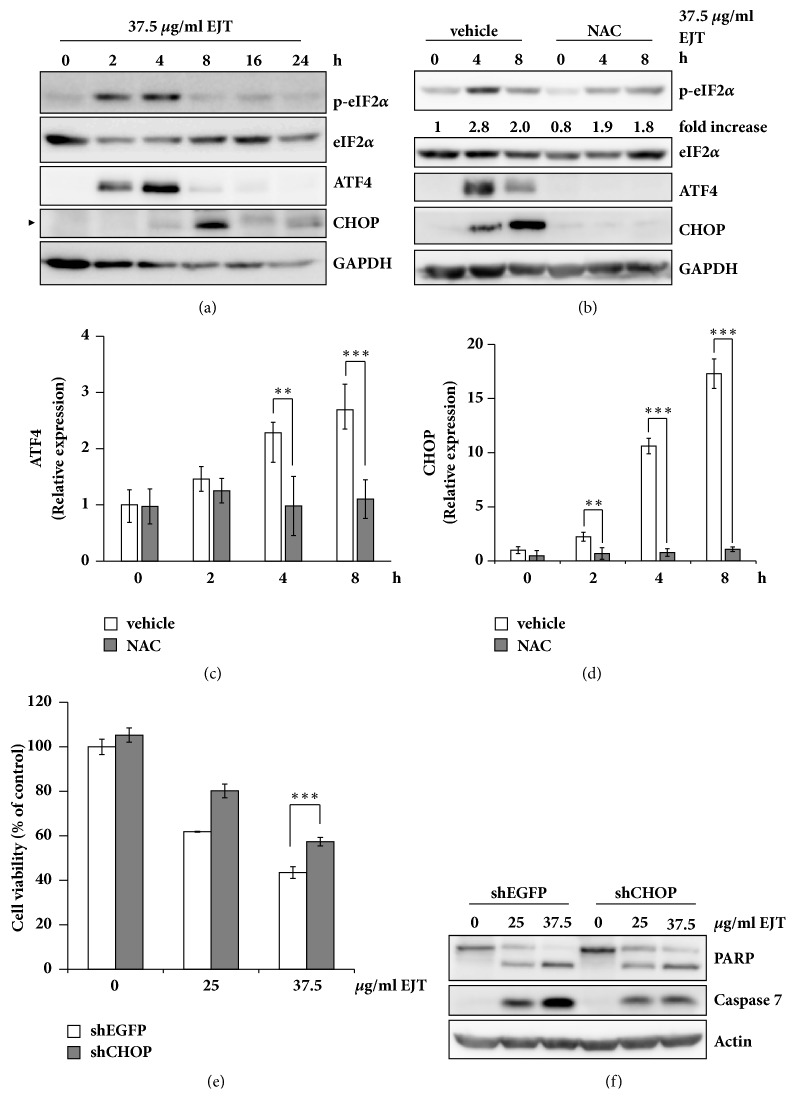
CHOP was involved in EJT extract-mediated apoptosis in MH7A cells. (a) MH7A cells were pretreated with 37.5 *μ*g/ml EJT extract for the indicated time. The cell lysates were subjected to SDS-PAGE and analyzed by immunoblotting for antibodies specific to ATF4, CHOP, and GAPDH. (b) MH7A cells were pretreated with 5 mM NAC for 2 h and then treated with 37.5 *μ*g/ml EJT extract for the indicated time. Immunoblot analyses were performed using specific antibodies. The fold increase in p-eIF2*α* expression is the ratio of the p-eIF2*α* to eIF2*α*. (c, d) The relative mRNA levels of ATF4 and CHOP were measured by using real-time quantitative PCR. Significant differences were indicated by ^*∗∗*^p < 0.01 or ^*∗∗∗*^p < 0.001. (e) MH7A cells were transfected with EGFP- or CHOP-specific shRNA. The cells were treated with indicated concentrations of EJT extract for 24 h and an MTT assay was performed to determine cell viability. Significant differences were indicated by ^*∗∗∗*^p < 0.001. (f) The cell lysates were subjected to SDS-PAGE and analyzed by immunoblotting with specific antibodies.

We investigated the expression of ER stress target genes to obtain insights into the molecular mechanisms of the cytotoxicity induced by EJT extract. The results indicated that EJT extract increased the phosphorylation of eIF2*α* and the expression of ATF4 and CHOP (Figure 3(a)). In addition, we tested whether the increased ER stress markers were associated with ROS generation. Pretreatment of NAC significantly decreased the phosphorylation of eIF2*α* (Figure 3(b)). Also, pretreatment of NAC reduced the expression of ATF4 and CHOP induced by EJT extract at both transcriptional and translational levels (Figures 3(b)–3(d)). CHOP is known to be the key regulator of ER stress-mediated apoptosis [10]. To determine whether CHOP was involved with EJT extract-mediated apoptosis, we decreased the expression of CHOP by using specific shRNA (supplementary material, Figure S1). As shown by these results, the knockdown of CHOP slightly increased cell viability and reduced caspase-7 activation and PARP cleavage induced by EJT extract in MH7A cells (Figures 3(e) and 3(f)). These results indicated that EJT extract-induced ER stress through ROS generation and that CHOP was involved with EJT extract-mediated apoptosis in MH7A cells.

### 3.4. EJT Extract Decreases Inflammatory Response Induced by TNF-*α* Treatment in MH7A Cells

**Figure 4:**
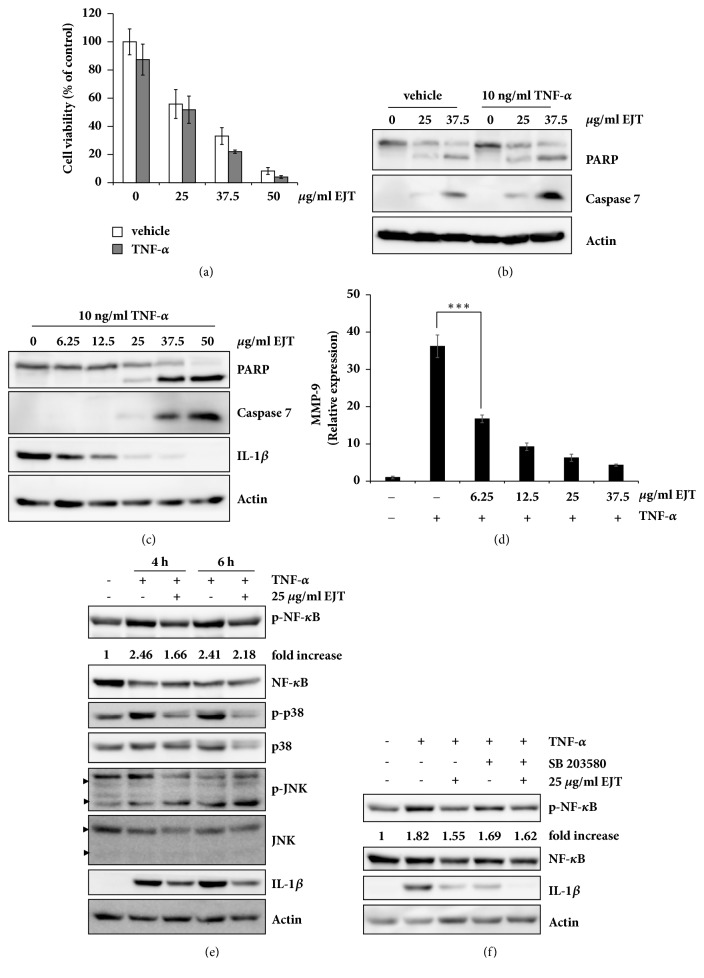
EJT extract significantly decreased TNF-*α*-induced inflammation in MH7A cells. (a) MH7A cells were pretreated with 10 ng/ml TNF-*α* or DMSO for 30 min and then treated with the indicated concentrations of EJT extract for 24 h. The cell viability was determined by using an MTT assay. (b) Immunoblot analyses were performed by using specific antibodies. (c) MH7A cells were pretreated with 10 ng/ml TNF-*α* for 30 min and then treated with the indicated concentrations of EJT extract for 24 h. The cell lysates were subjected to SDS-PAGE and analyzed by immunoblotting with antibodies specific to PARP, caspase 7, IL-1*β*, and actin. (d) The relative mRNA level of MMP-9 was measured by using real-time quantitative PCR. Significant differences were indicated by ^*∗∗∗*^p < 0.001. (e) MH7A cells were pretreated with 10 ng/ml TNF-*α* for 30 min and then treated with 25 *μ*g/ml EJT extract for indicated time. Immunoblot analyses were performed using specific antibodies. The fold increase in p-NF-*κ*B expression is the ratio of the p-NF-*κ*B to NF-*κ*B. (f) MH7A cells were pretreated with 20 *μ*M SB203580 for 2 h and then treated with 10 ng/ml TNF-*α*. After 30 min, MH7A cells were treated with 25 *μ*g/ml EJT for 6 h. Immunoblot analyses were performed using specific antibodies. The fold increase in p-NF-*κ*B expression is the ratio of the p-NF-*κ*B to NF-*κ*B.

To test whether the inflammatory conditions in MH7A cells affected the cytotoxic effects of EJT extract, MH7A cells were treated with EJT extract after 30 min of TNF-*α* pretreatment. Regardless of the TNF-*α* pretreatment, EJT extract treatment resulted in decreased cell viability and increased activation of caspase-7 and PARP cleavage in MH7A cells (Figures 4(a) and 4(b)). These results indicated that EJT extract treatment induced apoptosis in MH7A cells in inflammatory conditions. Oxidative stress is involved as an inflammatory mediator and can directly contribute to exacerbation of the disease symptoms of RA [23]. Therefore, we tested whether ROS induced by EJT extract treatment could affect the inflammatory response in MH7A cells. EJT extract treatment decreased IL-1*β* expression induced by TNF-*α* treatment, even at concentrations of 6.25–12.5 *μ*g/ml (Figure 4(c)). In addition, EJT extract treatment significantly reduced the mRNA expression of MMP-9 in MH7A cells (Figure 4(d)). To further clarify whether the anti-inflammatory effects of EJT extract were related to the regulation of the inflammatory signaling pathways, the effects of EJT extract on NF-*κ*B signaling were determined in MH7A cells. As shown Figure 4(e), EJT extract treatment downregulated the activation of NF-*κ*B in MH7A cells. In addition, EJT extract treatment reduced the phosphorylation of p38 induced by TNF-*α* treatment but did not affect the phosphorylation of JNK (Figure 4(e)). Therefore, we investigated whether the activation of p38 is related to anti-inflammatory effect of EJT in MH7A cells. As shown in Figure 4(f), pretreatment of SB203580, a specific p38 inhibitor, reduced the activation of NF-*κ*B and the expression of IL-1*β*. In addition, cotreatment of SB203580 and EJT significantly reduced the expression of IL-1*β* induced by TNF-*α* treatment in MH7A cells (Figure 4(f)). These results suggested that EJT extract induced apoptosis in MH7A cells, regardless of inflammatory conditions and decreased inflammatory response through the downregulation of p38 and NF-*κ*B activity.

## 4. Discussion

In this study, we found that EJT extract not only affected the abnormal proliferation and inflammation of RAFLS but also increased ROS through the induction of ER stress and apoptosis in MH7A cells. CHOP, the major regulator of the ER stress-mediated apoptosis, was involved in EJT extract-induced apoptosis. In addition, EJT extract treatment dose-dependently reduced the expression of MMP-9 and IL-1*β* induced by TNF-*α* pretreatment through the inhibition of NF-*κ*B activation in MH7A cells. These results suggested that EJT extract can be used as a drug supplement for RA to regulate the abnormal proliferation and inflammation of synoviocytes.

In this study, EJT extract induced ROS-dependent apoptosis in MH7A cells. The leaves and stems of* Eupatorium japonicum* are medicinal herbs used for the treatment various conditions, including diarrhea, vomiting, nausea, and indigestion [16]. The plant contains the essential oil thymol and pyrrolizidine alkaloids (PAs) [16, 17]. The PAs of EJT extract include indicine, amabiline, viridiflorine, and supinine [17]. Indicine N-oxide, derived from indicine, was reported to induce apoptosis through DNA damage and microtubule depolarization [21]. In addition, clivorine, a PA, was reported to increase ROS-mediated hepatotoxicity through a decrease in cellular GSH [24]. Many PAs have been established to cause hepatotoxicity, as mentioned above. Although clivorine exerts hepatotoxicity through oxidative stress, there is no report that clivorine exists in EJT. Furthermore, there have been no reports on the relationship between the PAs included in EJT and oxidative stress. Other studies have suggested that sesquiterpene lactone isolated from* E. japonicum* has apoptotic effects in many types of cancer [25]. Especially, Eupalinin A induced mitochondrial dysfunction and autophagic cell death in HL60 cells [26]. Collectively, since many bioactive molecules are contained in the EJT extract, it will be necessary to identify the precise molecular target(s) of the EJT extract that cause(s) cell death through ROS generation.

It is known that the pathway of NF-*κ*B activation is very complex and specific to environmental condition and cell types. The synovium of patients with RA contains many types of proinflammatory cytokines (such as TNF-*α*, IL-1*β*, and IL-6), chemokines, and MMPs (such as MMP-1, -3, and -9) [27]. These abovementioned factors are known to be regulated by NF-*κ*B [28, 29]. As NF-*κ*B is considered to be the master regulator of inflammatory cytokine production, there have been many efforts to develop a therapeutic agent capable of NF-*κ*B inhibition, but it is complicated by the complexity of NF-*κ*B activation and side effects [27, 30]. In our study, TNF-*α* pretreatment increased IL-1*β* expression, MMP-9 transcription, and NF-*κ*B activation, but cotreatment of the EJT extract reduced the factors mentioned above in a dose-dependent manner. These results were consistent with other study that* E. japonicum* extract decreased NF-*κ*B activation and expression of iNOS and COX-2 induced by Toll-like receptor agonists [31]. It is reported that activated p38 MAPK dominated in the synovial lining layer and in endothelial cells in synovial tissue from RA [32]. The p38 MAPK is considered as an attractive therapeutic target in RA because it plays an important role in the expression of proinflammatory cytokines [33]. Inhibition of p38 decreases not only the production of inflammatory cytokines such as IL-6 and IL-8 in rheumatoid synovial fibroblast but also reduces both synovial inflammation and the bone destruction by decreasing the generation of osteoclasts [34, 35]. The role of p38 MAPK in NF-*κ*B activation has been reported to be dependent on cell type- or stimuli-specific [36, 37]. In our study, pretreatment of SB203580 reduced NF-*κ*B activation and IL-*β* expression as similarly to EJT extract treatment. However, the cotreatment of EJT extract and SB203580 did not significantly reduce the activation of NF-*κ*B, unlike the clear reduction of IL-1*β* expression. These results suggested that EJT extract treatment had anti-inflammatory effects through inhibition of activation of p38 and NF-*κ*B. Therefore, we suggested that* E. japonicum *extract can be used as a therapeutic agent for RA treatment owing to its anti-inflammatory effects; however, it is necessary to elucidate the mechanism of NF-*κ*B and p38 inhibition to solve the problems in the development of such therapeutic agents.

In the synovium of patients with RA, increased ROS is one of the markers of RA. Many studies have suggested that positive correlation exists between oxidative stress and the pathology of RA [23]. Excessive ROS induces cartilage destruction, lipid peroxidation, DNA damage, and inflammation. Therefore, substantial evidence has suggested that antioxidants considerably ameliorate painful joints [23, 38, 39]. In addition, thymoquinone reduces osteolysis through a decrease in RANKL-induced ROS generation and the inhibition of MAPK and NF-*κ*B activation [40]. As described above,* E. japonicum* also contains the essential oil thymol [16]. Thymol is a polyphenolic compound found in thyme and it is known that the plant extracts, containing thymol, have anti-inflammatory and antioxidation effects. Yu et al. reported that thymol reduced high-fat diet-induced inflammation through a decrease in inflammatory cytokines, such as TNF-*α*, IL-1*β*, IL-6, and MMP-9 [41]. Thymol is also known to have an antioxidant activity, which eliminates the proxyl radicals and inhibits H_2_O_2_-induced apoptosis [42]. Interestingly, thymol has antioxidant effects at low concentrations but paradoxically induces prooxidant activity at high concentration, such as the increase of 8-hydroxy deoxyguanosine (8-OHdG) and membrane damage [43, 44]. Taken together, our results showed that unlike previously reports, EJT extract increased ROS generation but interestingly reduced the inflammatory response. Therefore, we intended to determine whether the apoptotic and anti-inflammatory effects of EJT extract are due to previously reported components, such as thymol or other components in EJT extract.

In our study, EJT extract increased the expression of the ER stress-related genes, ATF4 and CHOP, in MH7A cells; moreover, pretreatment of NAC significantly decreased the expression of these genes. The relationship between ER stress and oxidative stress is closely related to many physiological and pathological conditions; similarly, these stressors can affect the homeostasis of each. Therefore, many studies had been reported that increased ROS induced ER stress [45–48]. Lin et al. reported that protodioscin, a steroidal saponin, induced ROS-mediated ER stress and apoptosis in human cervical cancer cells. Interestingly, increased ER stress and apoptosis were attenuated by cotreatment with NAC, a JNK inhibitor or a p38 inhibitor, suggesting that ER stress was induced by ROS-mediated JNK/p38 activation [49]. In addition, ROS induces greater Ca^2+^ release in the ER by opening the inositol-1,4,5-trisphosphate receptor (IP_3_R) and ryanodine receptors, which consequently increases ER stress [50]. Panaxydol increases the cytosolic Ca^2+^ level through PLC*γ*-mediated Ca^2+^ release via the IP_3_R and ryanodine receptors. Increased cytosolic Ca^2+^ increases ROS production through CaMKII-TAK1-p38/JNK-NADPH oxidase activation, which results in ROS-mediated ER stress and apoptosis [51]. It was reported that thymol treatment increased intracellular Ca^2+^ level through extracellular Ca^2+^ influx and the release of Ca^2+^ from ER. Ca^2+^ release from ER after thymol treatment was associated with IP_3_R activation; however, increased Ca^2+^ was independent of thymol-induced apoptosis [52]. These previous studies may explain the observation of ROS-mediated ER stress in our study, but it will be necessary to elucidate the underlying mechanisms of EJT extract-induced ROS and apoptosis.

In our study, EJT extract treatment increased the expression of ER stress related to ATF4 and CHOP, and the expression of CHOP was associated with EJT extract-induced apoptosis in MH7A cells. These results were consistent with our previous studies in MH7A cells of apoptosis induced by hemp seed oil (HO) or chalcone-derived DK-59 treatment and the apoptotic role of CHOP [53, 54]. CHOP is known to be the major regulator of the apoptotic pathway under ER stress conditions. The overexpression of CHOP reduced the expression of the antiapoptotic factor Bcl-2 and, conversely, increased the expression of proapoptotic factors Bax and Bim, thereby increasing the mitochondrial Ca^2+^ level and ROS production [11, 51]. Our results were consistent with other studies, in which CHOP was associated with apoptotic effects; however, it is necessary to clarify the downstream target of CHOP to elucidate the mechanisms of EJT extract-mediated apoptosis.

## 5. Conclusions

In this study, we sought a therapeutic agent for RA from a natural product library. We found that EJT extract resulted in dose-dependent anti-inflammatory effects through inhibition of NF-*κ*B activation. In addition, at high concentrations, EJT extract induced ROS-dependent apoptosis without an increase in the inflammatory response. Increased ROS also induced the ER stress target genes ATF4 and CHOP, with CHOP involved in EJT extract-induced apoptosis. These results suggested that EJT extract may be effective as an RA drug supplement through the reduction of the inflammatory response and the abnormal proliferation of synoviocytes.

## Data Availability

The data used to support the findings of this study are available from the corresponding author upon request.

## Supplementary Materials

Supplementary Materials Figure S1: Knockdown of CHOP using specific shRNA decreased the expression of CHOP induced by Thapsigargin treatment. (a) MH7A cells were transfected with EGFP- or CHOP-specific shRNA. The cells were treated with 200 nM Thapsigargin for 8 h. Immunoblot analyses were performed using specific antibodies.
