# Supplementary material for: Apoptotic and Anti-Inflammatory Effects of Eupatorium japonicum Thunb. in Rheumatoid Arthritis Fibroblast-Like Synoviocytes
Source: Biomed Res Int. 2018 Jul 9;2018:1383697. doi: 10.1155/2018/1383697 (PMC6077679; doi:10.1155/2018/1383697)
Supplement: Supplementary Materials — Figure S1: Knockdown of CHOP using specific shRNA decreased the expression of CHOP induced by Thapsigargin treatment. (a) MH7A cells were transfected with EGFP- or CHOP-specific shRNA. The cells were treated with 200 nM Thapsigargin for 8 h. Immunoblot analyses were performed using specific antibodies. [file 1383697.f1.pdf]

(a)

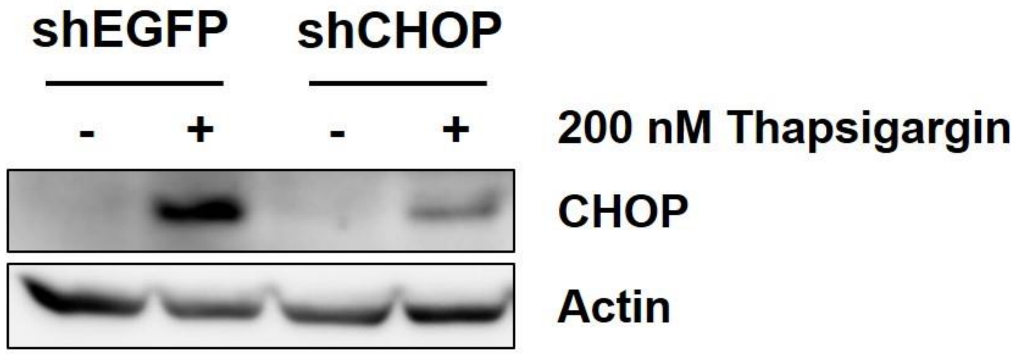


**Figure S1**. Knock-down of CHOP using specific shRNA decreased the expression of CHOP induced by Thapsigargin treatment. (a) MH7A cells were transfected with EGFP- or CHOP-specific shRNA. The cells were treated with 200 nM Thapsigargin for 8 h. Immunoblot analyses were performed using specific antibodies.
